# Prodrug Approach for Increasing Cellular Glutathione Levels

**DOI:** 10.3390/molecules15031242

**Published:** 2010-03-03

**Authors:** Ivana Cacciatore, Catia Cornacchia, Francesco Pinnen, Adriano Mollica, Antonio Di Stefano

**Affiliations:** Department of Drug Sciences, School of Pharmacy, “G. d’Annunzio” University, Via dei Vestini 31, 66100 Chieti, Italy

**Keywords:** glutathione, glutathione deficiency, glutathione prodrugs

## Abstract

Reduced glutathione (GSH) is the most abundant non-protein thiol in mammalian cells and the preferred substrate for several enzymes in xenobiotic metabolism and antioxidant defense. It plays an important role in many cellular processes, such as cell differentiation, proliferation and apoptosis. GSH deficiency has been observed in aging and in a wide range of pathologies, including neurodegenerative disorders and cystic fibrosis (CF), as well as in several viral infections. Use of GSH as a therapeutic agent is limited because of its unfavorable biochemical and pharmacokinetic properties. Several reports have provided evidence for the use of GSH prodrugs able to replenish intracellular GSH levels. This review discusses different strategies for increasing GSH levels by supplying reversible bioconjugates able to cross the cellular membrane more easily than GSH and to provide a source of thiols for GSH synthesis.

## 1. Introduction 

Glutathione (γ-L-glutamyl-L-cysteinylglycine, GSH), a water-soluble endogenous tripeptide, is the most abundant thiol present in mammalian cells, with concentrations up to 10 mM [1]. The liver, one of the tissues with the highest content of this tripeptide, is the main tissue participating in GSH biosynthesis [2]. Within the cell, GSH is kept in its thiol-reduced form (>98%) by glutathione disulfide (GSSG) reductase, an NADPH-dependent enzyme; additional amounts of GSH are present as glutathione disulfide (GSSG) and as glutathione conjugates (GS-R). Maintaining optimal GSH:GSSG ratios in the cell is critical to survival, since GSH is one of the primary endogenous antioxidant defense systems in the brain, removing hydrogen- and lipid-peroxides [3].

**Scheme 1:**
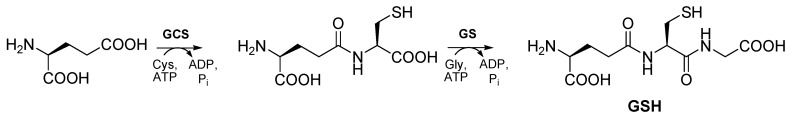
Biosynthesis of GSH.

GSH is synthesized *in vivo* by the consecutive action of two cytosolic enzymes in two ATP-dependent reactions (Scheme 1). In the first step, γ-glutamylcysteine synthetase (GCS) catalyzes the reaction between the γ-carboxyl group of glutamate and the amino group of cysteine (Cys) to form a peptidic γ-linkage, which protects GSH from hydrolysis by intracellular peptidases [1]. The second step is catalyzed by GSH synthetase (GS), which links the amine residue of glycine to the cysteine carboxyl group of γ-glutamylcysteine dipeptide to form GSH. The balance of cellular synthesis and consumption of GSH is regulated by feedback inhibition of the GCS reaction by the endproduct GSH [4,5].

Once intracellular GSH is produced, some of it is delivered to specific compartments, including mitochondria, endoplasmatic reticulum, and nucleus, but most is located in the extracellular spaces of the tissues [6]. Unlike intracellular GSH synthesis, GSH degradation occurs exclusively in the extracellular spaces of cells that express γ-glutamyl transpeptidase (γ-GT). This heterodimeric glycoprotein catalyzes the hydrolysis and transpeptidation of the GSH γ-glutamyl group [7]. The γ-glutamyl amino acids formed during this reaction are substrates of γ-glutamyl cyclotransferase, which converts them into 5-oxoproline and corresponding amino acids. The ATP-dependent conversion of 5-oxoproline to L-glutamate is catalyzed by the intracellular enzyme 5-oxoprolinase. Cysteinylglycine, formed in transpeptidation reaction, is split by dipeptidases to glycine and cysteine [8]. All these reactions constitute the γ-glutamyl cycle that can supply again amino acid precursors for additional GSH synthesis [8,9].

This review reports different strategies for increasing GSH levels by supplying prodrugs able to cross the cellular membrane more easily than GSH and to provide a source of thiols for GSH synthesis.

## 2. Biological Functions of GSH

**Figure 1:**
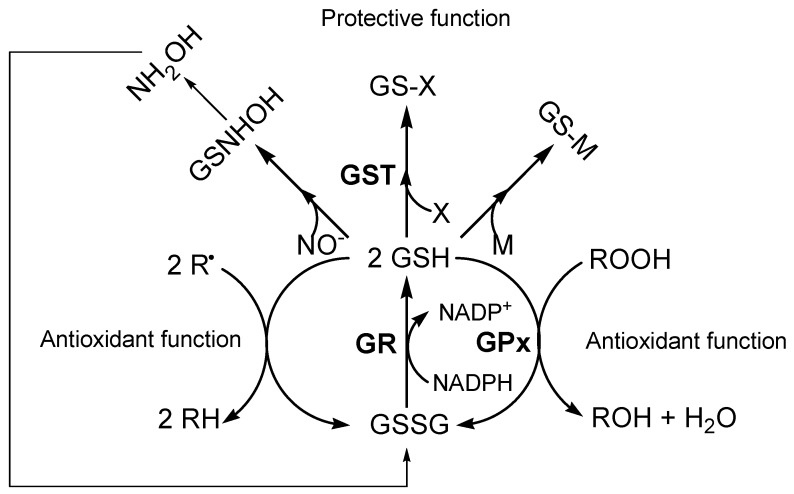
Biological functions of GSH.

GSH is involved in numerous biologically important processes. In particular, the key functional element of the GSH molecule is the sulfhydryl group of cysteine, which is involved in reduction and conjugation reactions that are usually considered the most important functions of GSH [10]. As an important antioxidant, GSH participates non-enzimatically and enzymatically in the protection against toxic compounds [11]. GSH protects the cell against the effects of free radicals (R^•^) and other reactive oxygen species (ROS) (e.g., hydroxyl radical, lipid peroxyl radical, peroxynitrite and H_2_O_2_) that may be formed normally in metabolism. In particular, GSH is the electron donor in the enzymatic reduction of both H_2_O_2_ and organic peroxides (ROOH) catalyzed by GSH peroxidase (GPx). GSSG produced in turn is rapidly reduced back to GSH by NADPH-dependent glutathione reductase (GR) (Figure 1) [12,13]. In the detoxification mechanism, GSH reacts enzymatically with a variety of xenobiotic electrophilic compounds in the catalytic reaction of glutathione-*S*-transferase (GST). The most common reactions involve nucleophilic attack by GSH on an electrophilic carbon of saturated, unsaturated or aromatic carbon atoms [10]. The resulting GSH S-conjugates are metabolized by the same enzyme of GSH degradation to glutamate, glycine and cysteine. While glutamate and glycine can be reabsorbed into the cell, the cysteine *S*-conjugates can be acetylated on the amino group of the cysteinyl residue by intracellular *N*-acetyltransferases to form the corresponding mercapturic acids. These acids are then released into the circulation and delivered to the kidney for excretion in urine, or they may undergo further metabolism [14]. Moreover, GSH plays an important role in metal (M) transport, storage and metabolism, and is involved in the process of conjugation of metals through production of complexes *via* non-enzymatic reactions [9,15]. GSH functions as a source of cysteine for metal binding, and as a reductant or cofactor in redox reactions involving metals. The sulfhydryl group of the cysteine moiety of GSH has a high affinity for metals such as mercury, silver, cadmium, lead, zinc and copper, forming a thermodynamically stable complex that can be eliminated [16].

Above all, GSH is implicated in the conjugation of nitric oxide (NO). In fact, intracellular GSH concentrations appear to play an important factor in regulating the susceptibility of the cell to NO and its derivates [17]. GSH reacts with NO to form hydroxylamine and GSSG. Nitric oxide readily reacts with superoxide to form peroxynitrite (ONOO^-^) and the latter will also react with GSH, leading to GSSG. If GSH levels are compromised due to oxidative stress conditions, neurons become particularly sensitive to NO and ONOO^-^ [18]. Peroxynitrite has been shown to promote the nitration of tyrosine residues to form the stable compound 3-nitrotyrosine; these NO-derived species are significant contributors to neuron degeneration.

Finally, GSH serves as substrate for formaldehyde dehydrogenase, which converts formaldehyde and GSH to *S*-formylglutathione. GSH is also involved in the glyoxalase system and in glutathionylation of proteins [19,20].

## 3. Diseases Associated with Altered GSH Metabolism

The importance of GSH in human disease is seen in a multitude of pathologies in individuals with inborn deficiencies of specific enzymes of GSH metabolism [7]. Patients with moderate GS deficiency showed hemolytic anemia and metabolic acidosis, while patients with a more severe insufficiency also developed 5-oxoprolinuria, bacterial infections and progressive dysfunction of central nervous system (CNS) [21]. Hemolytic anemia, and in some cases, neurological symptoms, were also observed in patients with deficiency of GCS, the first enzyme in GSH synthesis [22,23]. In humans, an inherited deficiency of the enzymes of the γ-glutamyl cycle leads to reduced production of GSH, and therefore the multiple roles of GSH could be reduced or absent in normal tissue. Accordingly, low GSH levels are frequently associated with mitochondrial dysfunction, activation of apoptosis through mitochondrial pathways, and production of sphingomyelin metabolites that can signal apoptosis [24]. Furthermore, the depletion of this powerful antioxidant renders cells particularly vulnerable to oxidative stress. The resulting damage is the key step in the onset and progression of many disease states. In fact, reduced GSH levels have been demonstrated to be a common feature of aging as well as of a wide range of pathologies, including neurodegenerative disorders, cystic fibrosis (CF), and several viral infections [19,25].

### 3.1. Neurodegenerative diseases

Alzheimer’s disease (AD), Parkinson’s disease (PD), and amyotrophic lateral sclerosis (ALS), the most common neurodegenerative disorders, may share mitochondrial dysfunction with slow progressive neuronal death as a final common pathway. In particular, thiol oxidation and loss of mitochondrial complex I activity precede excitatory amino acid-mediated neurodegeneration [26]. Oxidative stress can cause cellular damage, and ROS oxidizes critical cellular components such as membrane lipids, proteins, and DNA, thereby inducing apoptosis. The brain is especially vulnerable to oxidative damage as a result of its high oxygen consumption rate, its abundant lipid content, and its relative paucity of antioxidant enzymes compared to other tissues [27].

Among all neurodegenerative diseases, evidence for a dysfunction in GSH metabolism is strongest in PD [28]. PD is primarily associated with degeneration of the pigmented neurons in the substantia nigra pars compacta (SNpc), resulting in decreased dopamine (DA) nigrostriatal system availability and formation of Lewis bodies mainly composed of fibrillar α-synuclein [29,30]. Increased iron levels, inhibition of mitochondrial complex I activity, disappearance of melanin from the SN, and marked decrease of GSH in the SN suggest that oxidative stress plays a role in the initiation and/or progression of PD [31,32,33]. In particular, post mortem GSH levels in the SNpc of PD patients are remarkably lower than those of healthy subjects (40% to compared control subject) [34]. Although GSH is not the only antioxidant molecule reported to be altered in PD, it is hypothesised that the magnitude of its depletion is the earliest indicator of nigrostriatal degeneration; furthermore, striatal DA content and GSH levels are not altered in areas of the brain other than SNpc, or in other diseases affecting dopaminergic neurons [35,36,37,38]. Thus, it has been suggested that low levels of nigrostriatal GSH contents and consequent oxidative stress might contribute to the degeneration of dopaminergic neurons in idiopathic PD. 

While the role of GSH in the pathogenesis of PD is well documented, conflicting reports have been reported for AD. This disease is characterized by the loss of pyramidal neurons in the hippocampus and cortex as well as cholinergic neurons in the basal forebrain. The etiology of AD is not completely known, although there are different hallmarks that seem to play significant roles in the disease, such as β-amyloid (Aβ) deposits, τ-protein aggregation, oxidative damage in cell structures, and low levels of acetylcholine (ACh), [39]. Gu *et al*. [40] reported that GSH levels are depressed in AD cingulated cortex and AD substantia innominata, while Liu *et al*. [41] found these reduced levels only in red blood cells of male AD patients. Nevertheless, increased GSH levels have been observed by Adams *et al*. [42] in the midbrain and in the caudate nucleus, while normal GSH contents have been determined by Perry *et al*. [43]. Presumably, dissenting results are due to differences in techniques or difficulty in sample collection after death of AD patients. In any case, it has been determinated that GSH protects cultured neurons against oxidative damage resulting from β-peptide and 4-hydroxynonenal (HNE), a lipid peroxidation product that is increased in AD [44]. A significant decrease in Cu, and significant increases in Zn and Fe were found in AD hippocampus and amygdale, while Cu, Fe and Zn are elevated in senile plaques of AD. These metal ions can catalyze free radical reactions and contribute to oxidative damage observed in AD brain [17]. GSH protects these areas through formation of metal complexes *via* non-enzymatic reactions and may also be beneficial in normalizing the adverse effects of iron accumulation in the aging brain. [16].

### 3.2. Cystic fibrosis

Cystic fibrosis is a genetic disorder characterized by severe lung dysfunction due to an alteration in an ion transport protein, CF transmembrane conductance regulator (CFTR) [19]. CFTR maintains a cellular balance of ions such as sodium and chloride, and facilitates the trans-membrane export of the small organic anionic peptide GSH [45]. The latter serves as an essential antioxidant for protecting lung tissue from inhaled toxins and maintaining surfactant production. The lung is particularly vulnerable to oxidative attack from inhalation of pure oxygen, airborne toxins, and ROS released by lung phagocytes. GSH and GSH-associated enzymes present in the lung epithelial lining fluid (ELF) of the lower respiratory tract may be the first line of defense [46]. Patients with CF have decreased GSH levels in ELF (about 5-10% of normal) and blood plasma (about 50% of normal) [47,48]. In this disorder, GSH deficiency has also been associated with an increase in transcription of nuclear factor-kβ (NF-kβ), which participates in the regulation of the inflammatory cytokines [48]. Consequently, low GSH levels lead to inflammation and oxidative stress that damage cell membranes, proteins and DNA. In fact, individuals affected by CF frequently have higher levels of lipid peroxidation byproducts [49,50].

In addition to CF, several lung diseases, such as idiopathic pulmonary fibrosis, acute respiratory distress syndrome (ARDS), neonatal lung damage, and asthma are associated with low GSH levels [51].

### 3.3. Viral infection

Viral infection is often associated with redox changes characteristic of oxidative stress [52,53]. Several viral infections are characterized by marked depletion of extra- and intracellular GSH levels; the major viruses involved are human immunodeficiency virus (HIV), influenza, parainfluenza, rhinovirus, hepatitis C virus (HCV), and herpes simplex virus-1 (HSV-1) [54]. In particular, cultured cells infected with herpes simplex virus type 1, Sendai virus, and HIV have increased production of ROS, oxidation of the cellular GSH pool, as well as decreased intracellular GSH [55]. Moreover, GSH levels are reduced in plasma, ELF, peripheral blood mononuclear cells and monocytes in both seropositive HIV-infected individuals and AIDS patients, a deficiency that seems to correlate with disease morbidity [56]. Reduced GSH levels in plasma of HIV-infected patients could be due to either increased consumption of GSH or decreased availability of precursor amino acids, particularly Cys, which is a rate-limiting step in GSH synthesis [57]. It is believed that low intracellular GSH levels may facilitate the course of viral infections either by increasing viral replication or activating transcription factors such as AP-1 and NF-kB with consequent increased production of inflammatory cytokines (tumor necrosis factor, TNF-α), interleukin-1, and interleukin-6. Stimulation of cell lines with these cytokines produces oxidants and consumes intracellular GSH, resulting in appearance of immune deficiency, loss of CD4^+^ T-cells, metabolic disorders, inflammatory stress, and neurological deficiencies. [58].

### 3.4. Aging

The aging process involves various morphological and biochemical changes that occur from maturity to senescence, rendering the organism more vulnerable to disease and toxicity. Emerging evidence suggests that the free radical theory of aging seems to best explain these phenomena [59]. It proposes that there is an increase of oxidative damage with aging, which is the primary cause of the age-related declines in cellular function. In particular, aging results in increased formation and release of ROS, increased protein oxidation, reduced mitochondrial function, and DNA damage [26]. Increased lipid peroxidation products due to aging in rat brain have confirmed the involvement of oxidative stress in animal models [60]. Since GSH plays a central role in maintaining the oxidative balance of cells, a number of studies have analyzed GSH levels during aging [59]. One of these, by Liu *et al*. found age-dependent decline in GSH content in Fisher 344 rats [61]. They reported that GSH depletion was associated with a downregulation of GCS, the rate-limiting enzyme in *de novo* GSH synthesis. Further, with age, some tissues had decreased activity and mRNA content of GS, which catalyzes the second step in *de novo* GSH synthesis.

It has been observed that GSH levels are compromised in age-related nuclear cataracts, glaucoma, and macular degeneration (ARMD) [62]. Samiec *et al.* [63] determined plasma GSH levels in patients with retinopathies associated with two common age-related diseases, ARMD, and diabetes, and their results suggest that oxidation of GSH in specific pathologies such as diabetes and a decline in pool size may be important feature. Presumably, differences in GSH content in aging and/or diabetes or ARMD could be due to increased γ-GT activity, decreased GSH release from tissues consequent to decreased intracellular concentrations, or decreased GSH transporter functions with aging [61].

## 4. GSH Prodrugs

The contribution of GSH deficiency in many pathologies has stimulated a number of researchers to find new potential approaches for maintaining or restoring GSH levels in these patients. Currently, the use of GSH as a therapeutic agent is limited by its unfavourable biochemical and pharmacokinetic properties. GSH has a short life in human plasma (<3 min) and difficulty in crossing cell membranes, so administration of high doses is necessary to reach a therapeutic value [2,64]. In the effort to improve bioavailability, the prodrug approach appears to be the most promising, and some GSH prodrugs have been prepared in the attempt to solve these problems [65]. 

### 4.1. GSH esters 

**Figure 2:**
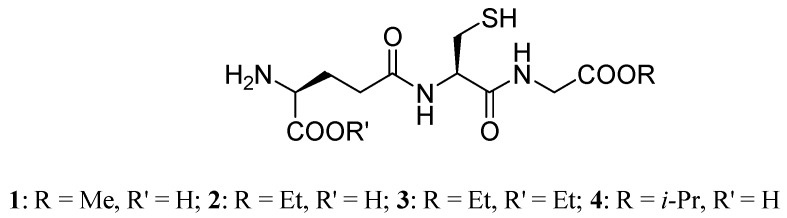
GSH ester prodrugs.

The direct supplementation of the cellular pool of GSH by GSH ester prodrugs is a useful strategy because it may circumvent the requirement for ATP and by-pass the GCS step in the *de novo* synthesis of GSH, which is feedback inhibited by GSH [66]. Esters of GSH can increase cellular GSH in a variety of tissue types and are particularly effective in restoring mitochondrial GSH [67,68,69]. Starting from these considerations, researchers have synthesized prodrugs of GSH, such as monomethyl (GSH-OMe, **1**), monoethyl (GEE, **2**), diethyl (GDE, **3**) and isopropyl (**4**) esters (Figure 2) [70]. GSH ester prodrugs **1**-**4**, in which the carboxyl group of the glycine residue is esterified, are thought to be more lipophilic than GSH , sufficiently so to be taken up intact by cells, and have been shown to rapidly raise intracellular GSH levels. In fact, prodrugs **1**-**4** are more effective than native GSH in enhancing intracellular GSH in human lymphoid cells, fibroblasts, endothelial cells, and rat hepatocytes *in vitro* [69,71,72]. As mentioned above, there is a known deficit of GSH in the SN of PD patients, so elevation of brain GSH levels may be an efficacious approach to neuroprotection in this disease. GSH is not able to elevate intracellular levels in neuronal cells, while the GEE prodrug evokes significant increase in GSH concentration. Subcutaneous administration of **2** (50 mg/kg/day) for 28 days is not capable of increasing brain tissue levels of GSH; central delivery of GEE is required. The consequent GSH elevation provides neuroprotection against oxidative stress or chronic mitochondrial impairment [73]. 

It is known that administration of GSH-OMe (**1**) is able to enhance liver and kidney GSH levels and prevent acetaminophen-induced hepatotoxicity in fasting adult mice [70]. For the first time, Chen *et al*. [74] demonstrated that GEE is an effective enhancer of tissue sulfhydryl levels in mesencephalic culture of old mice (30-31 months) and may also be effective in preventing acetaminophen-induced hepatotoxicity in old animals as well as **1** does. Moreover, GEE may have an important role in cellular defenses against liposaccaride-induced liver damage in mice because it increases the hepatic antioxidant capacity, improves mitochondrial function, and increases the survival rate of the mice [75]. 

Reperfusion of ischemic liver is associated with an increase in mitochondria of markers of oxidative injury and a decrease of GSH content [76]. GEE protects against reperfusion injury, providing high concentrations of intra- and extracellular GSH, which improves cellular function and detoxifies ROS generated in the vascular compartment [77].

The effects of GEE were also examined on ischemic damage, using intracerebroventricular infusion of the agent into the third ventricle during 2 h middle cerebral artery occlusion and 48 h of reperfusion. GEE has a marked neuroprotective effect on tissue infarction induced by reversed focal cerebral ischaemia and has increased cytoplasmatic GSH, thus promoting the ability of cells to resist ischemia-induced oxidative stress [78]. Furthermore, treatment with GEE prodrug seems to protect against toxicity caused by heavy metals (*i.e.* mercuric and cadmium ions), and antitumoral agents (*i.e.* cisplatin, cyclophosphamide, melphalan), both responsible for intracellular GSH depletion [9]. Finally, it has been recently demonstrated that GEE can suppress HIV expression in chronically infected monocytic cells at multiple stages of the virus activation processes [54].

**Figure 3:**
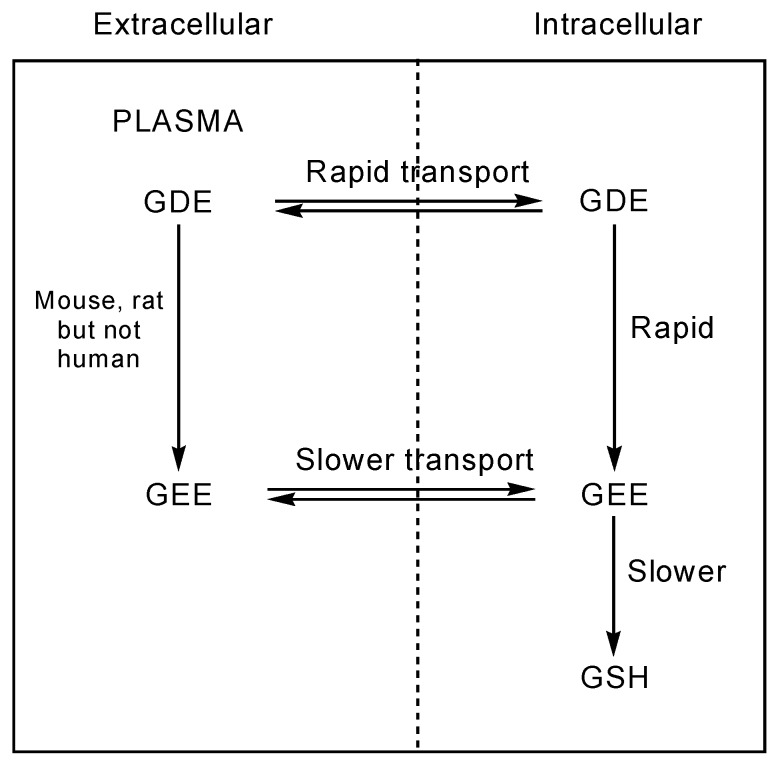
Transport and cleavage of GDE prodrug as proposed by Levy *et al*. [79].

Levy *et al*. [79] showed that human erythrocytes, lymphocytes, and ovarian tumor cells transport GDE (**3**) much more effectively than the corresponding GEE. Studies conducted on human erythrocytes have revealed that GDE is readily taken up by cells and is rapidly converted intracellularly to GEE, which does not efflux. Subsequent slower intracellular hydrolysis of GEE yields GSH. GDE is therefore able to increase liver GSH levels more effectively compared to the same dose of GEE in hamster liver (Figure 3) [5,79]. GDE releases two mols of ethanol compared to GEE, and thus the consequent toxicity should be increased. However, Anderson *et al*. [80] observed less toxicity with the GDE prodrug than with GEE in preliminary *in vivo* studies carried out in hamsters, probably due to an interaction of GDE with metal ions void of cellular toxicity. Additionally, the effect of GEE and GDE on the cellular concentration of GSH and cysteine in P388D_1_ macrophages *in vitro* was investigated by Minhas and Thornalley [81]. Incubation of P388D_1_ cells with GEE (1mM) gave a small increase in GSH levels while incubation with GDE resulted in a marked increase in these levels. This indicates that GDE is a much more effective vehicle for delivery of GSH into cells than GEE. Moreover, GDE also increases the cellular concentration of cysteine and γ-glutamylcysteine, suggesting that *de novo* synthesis of GSH is also stimulated [81]. 

Exogenous oxidative stress induced by *tert*-butyl hydroperoxide accelerates lipid peroxidation of cultured gastric mucosal cells. It has been demonstrated that GSH isopropyl ester (**4**) renders cells more resistant to *tert*-butyl hydroperoxide through the prevention of lipid peroxidation [82].

Gastric epithelial uptake of **4** seems identical to that of native GSH by the cells and distinctly different from that observed in other cells types, such as lymphoid and endothelial cells, fibroblasts and hepatocytes [69,71,72]. Furthermore, GSH isopropyl ester prodrug protects against ischemia damage in rat brain and may protect against the effects of hypoxia/hyperglycemia in rat brain hippocampus [83,84]. 

### 4.2. Cysteinyl-modified GSH derivatives

**Figure 4:**
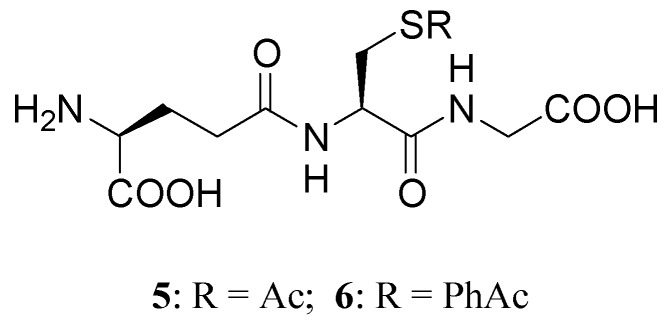
S-acyl-GSH prodrugs.

Another approach to replenish the intracellular GSH stores is possible by using *S*-acyl prodrugs of GSH, such as *S*-acetyl- (SAG, **5**) or *S*-phenylacetyl- (**6**) GSH prodrugs (Figure 4) [85]. *S*-acyl GSH prodrugs are capable of crossing the cell membrane and can be recognized as substrates by γ-GT. In particular, a good increase of intracellular GSH was observed after incubating rat brain synaptosomes with SAG for 30 min, while incubation with *S*-phenylacetyl GSH prodrug [85] caused less of an increase. Moreover, SAG is able to increase intracellular SH groups as reported by Vogel *et al*. [86], is more stable in blood plasma than GSH, and enters the cells directly, where it is converted to GSH by the abundant cytoplasmatic thioesterases. 

As mentioned above, decreases in GSH have been described in peripheral blood mononuclear cells and in red blood cells isolated from HIV-infected subjects [56]. SAG is significantly more efficient as a GSH-replenishment agent in both mock-infected and HSV-1 infected cells. Moreover, Vogel *et al*. [86] demonstrated that only systemic SAG (6.25 μg/g body weight per day) significantly avoided HSV-1 induced mortality in hr/hr mice. In contrast, systemic GSH given at the same concentrations exhibited no protective effect. Therefore SAG is a suitable antiviral agent against HSV-1 both *in vitro* and *in vivo*, and may be of benefit in the therapy of HSV-1 infections [54,86,87].

**Figure 5:**
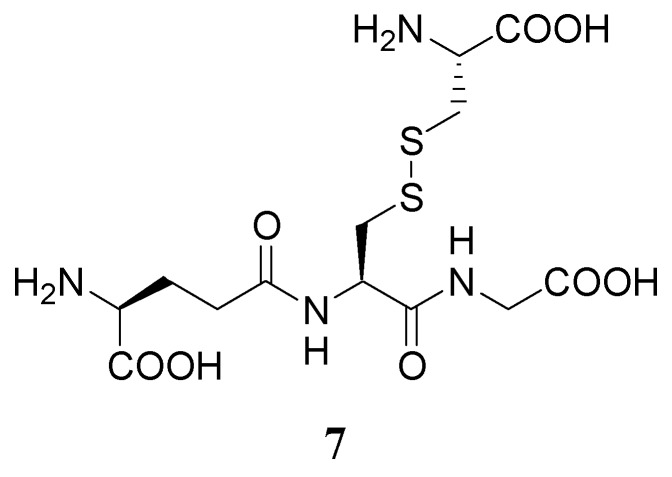
L-CySSG prodrug.

Another GSH prodrug under investigation is L-cysteine-glutathione mixed disulfide (L-CySSG, **7**) has been investigated as a protective agent against xenobiotic-induced hepatotoxicity (Figure 5) [88]. L-CySSG can be considered a sulfhydryl-modified prodrug of GSH, in which reactive thiol groups of GSH and cysteine are masked in a disulfide linkage. L-CySSG is a highly effective liver protective agent to prevent acetaminophen hepatotoxicity. Its protective functions are remarkable compared to GEE and to the cysteine prodrug *S*-(2-hydroxymethylmercapto)-L-cysteine. The efficacy of L-CySSG in protecting mice against ACP toxicity is in contrast to the lack of protection observed with D-CySSG, suggesting that GSH is released from L-CySSG. 

**Figure 6:**
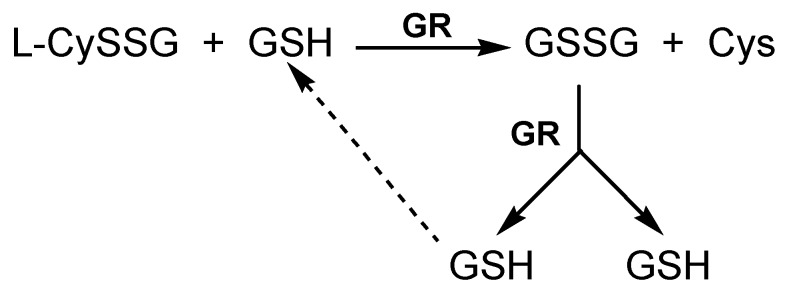
Disulfide bond reduction of L-CySSG prodrug as proposed by Berkeley *et al*. [88].

This could be the consequence of an enzymatic reduction of the disulfide bond, or from the GSH-dependent thiol disulfide exchange reaction with L-CySSG catalyzed by GR. Thus, L-CySSG provides not only GSH itself, but also the key aminoacid for *de novo* GSH biosynthesis (Figure 6) [88].

L-CySSG prodrug was further investigated by Phimister *et al*. [89] in the attempt to deliver GSH to Clara cells during naphthalene (NA)-exposure. It substantially decreases the levels of protein adducts formed by reactive metabolites of NA, and is able to limit GSH loss caused by NA when administered *in vivo*. Although its mechanism of action is still unclear, L-CySSG may be a good therapeutic tool for treatments involving supplementation to the thiol pool due its efficacy and chemical stability [89].

### 4.3. Cysteine prodrugs

Under physiological conditions, the cellular availability of Cys is considered to be the rate-limiting factor in the synthesis of GSH [90]. As a consequence, compounds that can be metabolized to Cys could be used as prodrugs to increase neuronal GSH levels. Cysteine could not be used *per se* because it is toxic in high concentrations and because it easily oxidizes to cystine, a more insoluble compound, making a stable formulation difficult to prepare. 

*N*-acetylcysteine (NAC) is the simplest Cys prodrug that can be systemically administered to deliver cysteine to the brain [91,92,93]. NAC is a good source of thiol groups able to stimulate GSH synthesis, to promote detoxification and to act as direct scavenger of ROS [94]. NAC was clinically used as a mucolytic agent in pulmonary diseases and it has been reported to have beneficial effects in the treatment of age-related neurodegenerative diseases and in conditions associated with GSH deficiency, including acetaminophen-induced hepatotoxicity, HIV infection, cystic fibrosis, and diabetes [95,96,97,98]. Its use, however, has been limited by several drawbacks, including low membrane penetration and low systemic bioavailability [99,100]. In the attempt to ameliorate these problems, an amide (*N*-acetylcysteine amide, NACA), and some esters of NAC were synthesized [101,102]. The studied esters included alkyl esters, glycolamide esters and acyloxymethyl esters formed at the carboxylic function, whereas the thiol group was protected as an *S*-benzoyl ester and an *S*-benzoylcarbamate ester [102]. Furthermore, *S*-allyl cysteine (SAC), *S*-methyl cysteine (SMC), *S*-ethyl cysteine (SEC) and *S*-propyl cysteine (SPC) are hydrophilic cysteine-containing compounds naturally formed in Alium plants such garlic and onion [103]. It has been demonstrated that the supplementation of SAC, SMC, SEC and SPC could increase GSH levels in plasma and organs and enhance antioxidant protection by elevating the GPx and SOD activity [104,105,106].

**Figure 7:**
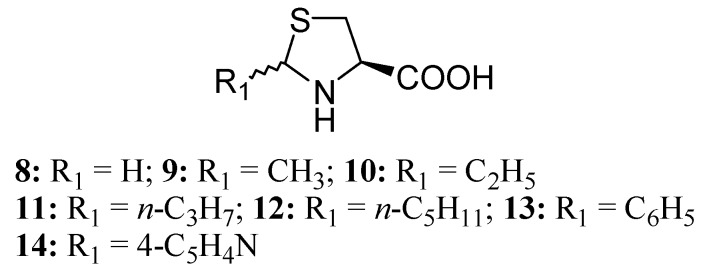
L-Thiazolidine-4-carboxylic acids.

Several Cys prodrugs with masked sulphydryl groups in the form of thiazolidine-4-carboxylic acids were synthesized from L-Cys and the corresponding aldheydes in order to avoid spontaneous oxidation to produce peroxides, or other reactions possible for Cys alone [107,108]. Among these, L-thiazolidine-4-carboxylic acid (TCA, **8**) is activated to Cys by proline oxidase, while 2-alkyl-substituted derivatives (**9**-**14**) spontaneously release Cys and its corresponding aldheyde (Figure 7).

**Figure 8:**
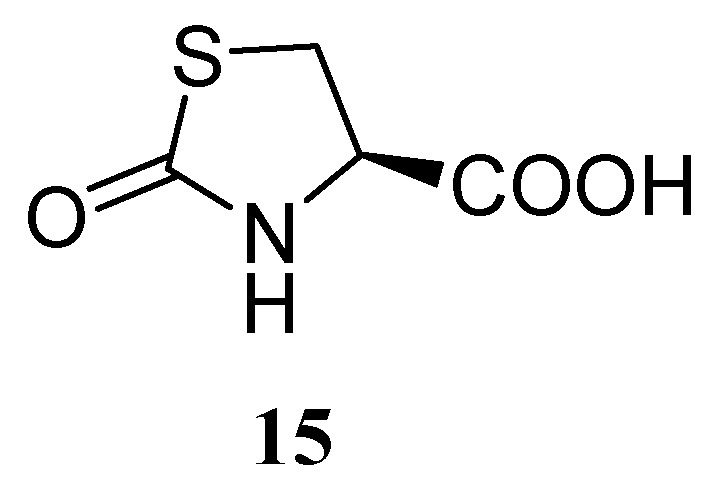
L-2-Oxothiazolidine-4-carboxylate.

L-2-Oxothiazolidine-4-carboxylate (OTC, **15**) (Figure 8) is the most studied molecule of this class as cysteine donor for the synthesis of GSH. OTC is intracellularly converted to Cys by the action of 5-oxoprolinase. Porta *et al*. [109] showed that OTC is able to increase the circulating concentration of Cys and the intracellular levels of Cys and GSH in lymphocytes.

In order to avoid any possible toxicity associated with the liberation *in vivo* of potentially toxic aldehydes, Roberts *et al*. [110] synthesized new 2-substituted-thiazolidine-4-carboxylic acids by condensation of L-Cys with naturally occurring aldose monosaccharides, such as glyceraldheyde, arabinose, lyxose, ribose, xylose, galactose, glucose and mannose. These prodrugs, when non-enzymatically degraded *in vivo*, liberated a non-toxic aldheyde and L-Cys, which could then act to elevate cellular GSH levels in the target tissue.

Methionine (Met) is one of the principal sources of organic sulphur for body processes and is considered a Cys prodrug [111]. Methionine supplementation increased tissue GSH levels following its intracellular conversion to Cys *via* the transulfuration pathway [2]. The transulfuration pathway converts Met to Cys, which is subsequently converted to GSH *via* the GSH synthetic pathway.

**Figure 9:**
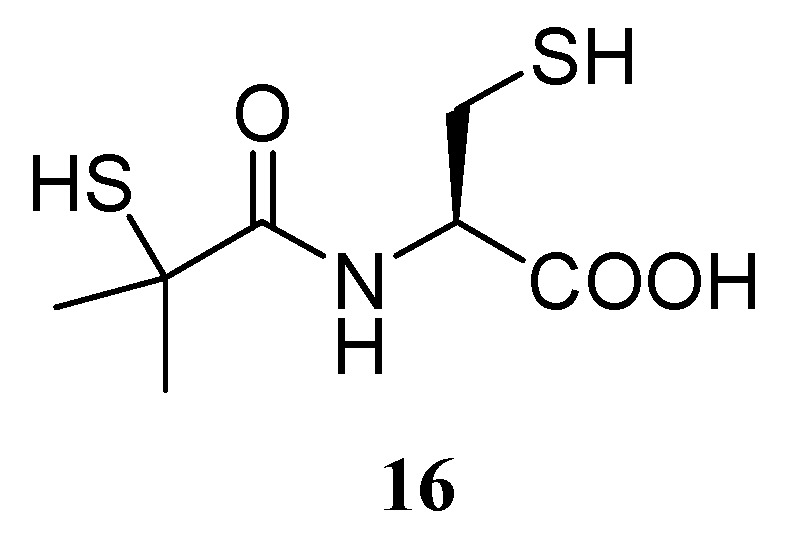
Chemical structure of bucillamine.

Under conditions of GSH depletion, the potential benefits of bucillamine (**16**) (Figure 9), a synthetic cysteine-derivative clinically used in Japan and Korea for the treatment of reumatoid arthritis, could also be considered [112].

This compound can be rapidly transported into cells by the same pathway utilized by Cys and can restore intracellular GSH levels, probably by donating thiol groups to GSH rather then *de novo* synthesizing GSH [113]. This hypothesis is supported by Amersi *et al*. [114], who demonstrated that the efficacy of bucillamine was correlated with a remarkable ability to enhance GSH levels in the liver and decrease levels of oxidated GSH in both the liver and blood perfusate in normal and steatotic livers.

## 5. GSH Codrugs

The codrug approach represents a new alternative strategy for enhancing physiological GSH levels or inhibiting its degradation. Two different synergistic drugs linked together *via* a readily cleavable covalent bond can regenerate the parent drugs at the desired site of action with improved physicochemical properties for drug delivery, compared to those of the individual drug entities themselves [115,116,117].

**Figure 10:**
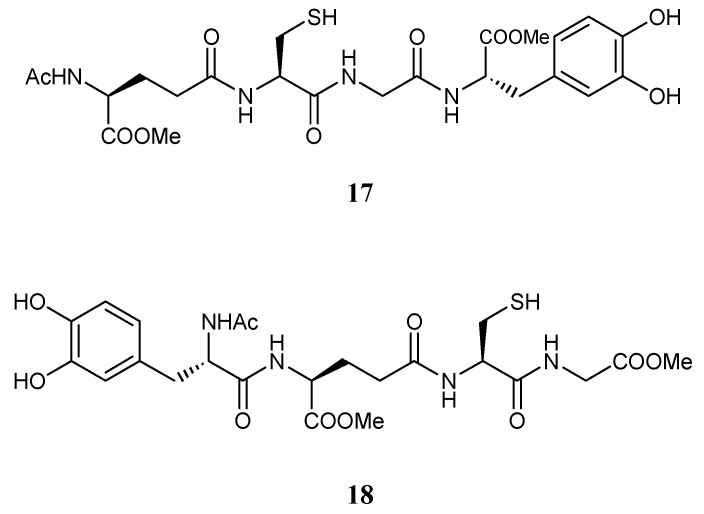
Multifunctional GSH codrugs.

Pinnen *et al*. [118] proposed the synthesis of reversible bioconjugates of GSH (**17**, **18**) in which LD is linked covalently *via* an amide bond to GSH in the C- and N-terminal position, respectively (Figure 10). These bioconjugates were able to prolong rat plasma LD levels and striatal DA concentration compared to an equimolar dose of LD, thus minimizing LD plasma fluctuations responsible for off/on effects.

Furthermore, compared to LD, they showed antioxidant properties, and thus may have neuroprotective potential in brain diseases marked by elevated free radical production. Codrugs **17** and **18** could represent useful new anti-Parkinson agents devoid of the pro-oxidant effects associated with LD therapy and potentially able to restore the GSH depletion noted in the SNpc of PD patients [118].

**Figure 11:**
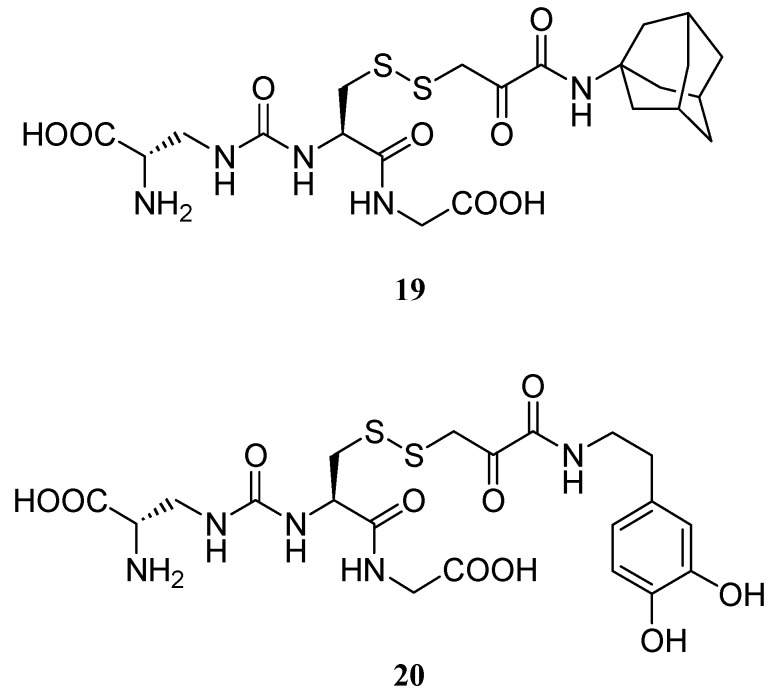
Glutathionyl conjugates.

Recently, More and Vince [119] developed a novel approach by which endogenous GSH transporters located on the luminal side of the blood-brain barrier (BBB) are exploited to deliver anti-Parkinson drugs that normally cannot cross the BBB, such as adamantamine and dopamine. The design of GSH transporter targeted conjugates (**19**, **20**) (Figure 11) required a carrier for the delivery to the BBB, the active drug, and an appropriate linker to conjugate the carrier with the molecule. 

A metabolically stable analogue of GSH containing urea in place of the γ-glutamyl-cysteinyl peptide bond was incorporated as a carrier molecule due to its known stability against γ-GT cleavage and maintenance of structural features necessary for recognition by GSH transporters [120,121]. A useful advantage of this carrier is also the antioxidant activity of the released GSH analogue that could attenuate the damage caused by oxidative stress during PD. Mercaptopyruvic acid was chosen as a suitable linker and was attached to the carrier *via* a heterodisulfide bond, due to the known stability of this linkage in plasma and the abundance of enzyme disulfide reductase in the brain compared to other body compartments [122]. The amide bond between the amine functional group of dopamine/adamantamine and the linker is expected to be cleaved by peptidases in the brain with reasonable stability in plasma.

Preliminary experiments successfully confirmed the carrier mediated-transport of conjugates **19** and **20** in an *in vitro* BBB model and their ability to release the active drug at the target site, thus representing an innovative approach for the targeted delivery of anti-Parkinson drugs into the CNS using the GSH transport system. 

**Figure 12:**
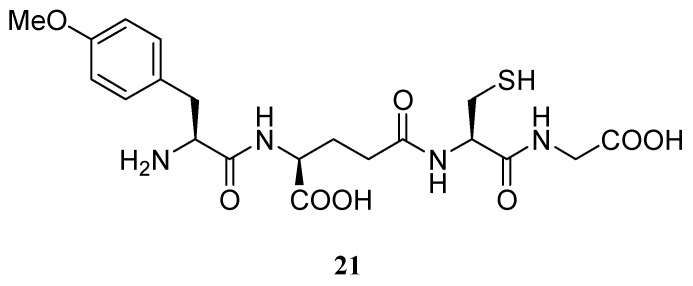
Structure of UPF1.

Recently, Ehrlich *et al*. [123] designed and synthesized a library of new GSH codrugs with hydroxyl radical scavenging ability. In particular, UPF1 (**21**), which contains two antioxidant molecules, as GSH and tyrosine (Figure 12), proved more hydrophobic than GSH and much better able to interact with plasma membrane and/or with hydrophobic binding sites of several proteins; its antioxidant activity was 60-fold higher than that of GSH [124].

Although the exact mechanisms of the protective action of UPF1 remained unclear, it has been demonstrated that UPF1 modulates the activity of G-proteins in the brain tissue and can act as a scavenger or a signal molecule increasing GSH levels or the GSH redox ratio [124], thus providing promising leads for design of powerful antioxidants for treatment of conditions associated with reduced GSH levels.

## 6. Other Strategies for Increasing GSH Levels

Given the many advantageous aspects of increased GSH levels, other strategies for elevating brain GSH content have been considered as potential therapeutic approaches useful in some of the disorders mentioned above.

Pileblad and Magnusson [125] reported that the intracerebroventricular administration of γ-glutamylcysteine (γ-GC), the limiting substrate in the synthesis of GSH, dose-dependently increases GSH levels (145–170% of control) in the substantia nigra and in the rest of the brain stem of rats. The ethyl ester of this dipeptide, (γ-glutamylcysteinylethyl ester, γ-GCEE), has been successively demonstrated to enhance the efficacy of γ-glutamylcysteine to cross the plasma membrane and to upregulate GSH biosynthesis [126]. In isolated rat hepatocytes, γ-GCEE is transported into liver cells more readily than GSH, hydrolyzed to γ-GC by esterase and then converted to GSH by GS [127]. γ-GCEE increased GSH levels in several different cell types and systems, such as ischemia-reperfusion in liver [128,129] and in heart [130,131], in carbon tetrachloride hepatic injury [132], and in selenium-deficient heart [133].

## 7. Conclusions

The powerful pleiotropic action of GSH makes this tripeptide an attractive tool for drug design. Reduced GSH levels have been widely documented in aging and in several pathological conditions such as neurodegenerative disorders, cystic fibrosis, and viral infections. Unfortunately, its unfavourable biochemical and pharmacokinetic properties limit the use of GSH as therapeutic agent. This review has investigated several strategies that could be used to treat a wide array of pathologies associated with GSH deficiency. Although the potential use of GSH prodrugs needs further exhaustive studies, they may offer a promising therapeutic alternative for reducing the GSH functional loss related to many human diseases.
